# Risk factors for adolescents’ mental health during the COVID-19 pandemic: a comparison between Wuhan and other urban areas in China

**DOI:** 10.1186/s12992-020-00627-7

**Published:** 2020-10-09

**Authors:** Shitao Chen, Zeyuan Cheng, Jing Wu

**Affiliations:** Faculty of Psychology, Beijing Normal University, Beijing, 100875 China

**Keywords:** Adolescents, Mental health, Risk factors, COVID-19

## Abstract

**Background:**

The outbreak of Coronavirus Disease is causing considerable acute risk to public health and might also have an unanticipated impact on the mental health of children and adolescents in the long run. This study collected data during the national lockdown period in China and aims to understand whether there is a clinically significant difference in anxiety, depression, and parental rearing style when comparing adolescents from Wuhan and other cities in China. This study also intends to examine whether gender, grade in school, single child status, online learning participation, parents’ involvement in COVID-19 related work, and parents being quarantined or infected due to the disease would lead to clinically significant differences in anxiety and depression. Beyond that, this study explored the pathways among the different variables in order to better understand how these factors play a part in impacting adolescents’ mental health condition.

**Results:**

Results showed that there was a statistically significant difference in anxiety symptoms between participants who were from Wuhan compared to other urban areas, but not in depressive symptoms. In addition, participants’ grade level, gender, relative being infected, and study online have direct positive predictive value for depressive and anxiety symptoms, whereas location and sibling status have indirect predictive value. Having relatives who participated in COVID-19 related work only had positive direct predictive value toward depression, but not anxiety.

**Conclusions:**

This study discovered several risk factors for adolescents’ depression and anxiety during the pandemic. It also called for a greater awareness of Wuhan parents’ mental wellbeing and recommended a systematic approach for mental health prevention and intervention.

## Background

The outbreak of Coronavirus Disease is causing considerable acute risk to public health and is impacting the entire world. At the time of this writing, there are more than 5.4 million confirmed cases and 0.3 million people who have died as a consequence of COVID-19 [5]. To control the epidemic, different levels of control measures were applied nationwide, and the Chinese government has implemented the most severe restrictions to Wuhan, with a complete lockdown of the population starting from January 23, 2020 [31]. Other interventions were also implemented in China during the epidemic, such as nationwide traffic restrictions, home quarantine policies, and school lockdowns. This meant a huge and sudden change of regular life—people might experience sudden separation from loved ones, some encountered shortages of living supplies, and many people also faced great financial distress due to job closures and reduced wages [11].

While struggling under these special circumstances, the stringent restrictions greatly controlled the epidemic and protected people’s physical health, so that more attention shifted to the psychological field. Shortly after the outbreak of COVID-19, Qiu and her team [18] implemented an early nationwide survey to understand the level of psychological distress among the Chinese people. The resulting report presented the different types of psychological distress that people were facing, and also indicated that children and adolescents had the lowest levels of peritraumatic distress. However, children and adolescents are also vulnerable, and the results do not mean that this population is free from distress. Rather, the pre-existing “protective factors”, such as strict home quarantine which ensured a lower morbidity rate for children and adolescents, might have had an unanticipated negative impact on the mental health of children and adolescents in the long run.

The repercussions of COVID-19 on children and adolescents’ mental health are best conceptualized multidimensionally. First, the closure of school and community services, as well as limited social interaction, shortened the amount of daily activities in which children can engage [12]; it also disrupted their regular routine and made adjustment to a new routine challenging. Second, to keep up with their learning schedule, most of the schools moved the traditional class to an online setting. Virtual spaces, such as Dingding and Zoom, created a new form of learning, but also increased students’ access to electronic devices, which could have an impact on students’ mental and physical health. In addition, the home quarantine policy reorganized family dynamics. Parents not only have to work from home and deal with their economic and emotional distress, but also spend extra time in taking care of their children and dealing with their children’s emotions, such as isolation from peers and anxiety and depression. Research has shown that when parents experience elevated levels of cumulative stress, their parental behaviors might become more rigid and abusive [13, 29], which, in turn, would negatively impact children’s mental health [23, 30]. A recent study conducted in Singapore also supported these findings and found that parents’ perceived impact of COVID-19 is closely connected with their increased parenting stress, and the parental stress, in turn, increased the risk of harsh parenting [3].

Moreover, COVID-19 might influence children and adolescents’ adjustment in a cascading fashion, especially for children and adolescents whose family members are directly impacted by the pandemic. For example, if family members were infected, served as first-line medical staff, or died due to COVID-19, they might experience more severe mental health impacts than other youth. Furthermore, these deleterious effects could be synergistic, rather than just additive. Previous epidemics, such as Influenza A (H1N1), have made clear the connection between emotional distress (such as anxiety and depression) and viral diseases [4]. Therefore, we anticipate that COVID-19 might also influence children and adolescents’ mental health.

This study aims to understand Chinese adolescents’ mental health status during the COVID-19 period and examine whether there is a clinically significant difference in anxiety, depression, and parental rearing style when comparing adolescents from areas that have different levels of severity of COVID-19 (i.e., Wuhan vs. other cities in China). We hypothesized that there might be a significant difference in the variables stated above when comparing Wuhan and other urban areas in China, because a previous study has concluded that Wuhan adolescents’ depressive symptoms were significantly higher than other cities in Hubei Province (of which Wuhan is a part) [27].

This study also intends to examine whether gender, grade in school, single child status, online learning participation, parents’ involvement in COVID-19 related work, and parents being quarantined or infected due to the disease would demonstrate clinically significant differences in anxiety and depression. We hypothesized that there would be significant differences in anxiety and depression, but we are uncertain which factors would contribute to these differences.

Finally, this study intends to explore the pathways among the different variables and understand how these factors play a part in impacting adolescents’ mental health condition. Previous studies have revealed significant associations between family factors and changes in child mental health during the COVID-19 period across a number of countries, and parenting behaviors is one of the identified factors [24]. Anxious parental rearing style also proved to be a mediator between stressful life events and severity of children’s anxiety symptoms [16]. Furthermore, perceived parental rearing style appeared to be related to certain demographic factors, such as gender and grade [7]. Therefore, we hypothesized that parental rearing style, which is represented by parenting behaviors, might serve as a mediator between demographic variables (i.e., grade, location, gender, and sibling status) and adolescents’ mental health condition (i.e., anxiety and depression) under this stressful public health event (COVID-19).

## Methods

### Participants and sampling

This is a cross-sectional study, using data randomly collected in three cities, Wuhan, Beijing and Hangzhou, during the pandemic. The data collection started on February 22, 2020, and ended on March 8, 2020, a time period when Wuhan was under complete lockdown. During the recruiting process, the survey, titled “A Survey to Understand Children and Adolescents’ Mental Health Condition during the Pandemic”, was sent out to teachers, parents, and students in Wuhan, Beijing and Hangzhou through WeChat, a popular messaging and social media app in China. The survey consisted of a total of 63 items, and it was expected to take about 5 min to complete. We asked these initial contacts to pass this information along to people who meet the inclusion criteria in these cities. The inclusion criteria include: Students who are currently enrolled in grade 7 to 12, who accept the informed consent, and are willing to participate in this survey.

A total of 7866 adolescents in these three cities were recruited for the study. Deleting the data from protocols in which the adolescent answered in a total period of time less than 100 s (an average of approximately 3 s per question), a total of 7772 protocols were considered to be valid. Of the participants whose survey results were considered valid, 2850 were from the Wuhan area, and 4922 of them were from Beijing and Hangzhou. Approximately half of them were male (47.77%, *n* = 3713) and half were female (52.23%, *n* = 4059). Due to the similarity of Beijing and Hangzhou’s level of severity during COVID-19, this research combined these two areas together and considered them as “Other Urban Areas”, to be compared in the aggregate with Wuhan.

### Ethical approval and consent

Before beginning the survey, the participants were notified about the purpose and procedures of this study through Questionnaire Star, a survey platform used to collect information. Online informed written consent was obtained from all participants and their parents before they began to fill out the questionnaire. This study was reviewed and approved by the Committee for Ethical Affairs of Beijing Normal University, Faculty of Psychology.

### Measures

To understand the relationship between students’ mental health condition and their experience during the pandemic, the survey items consisted of some experimenter-generated items specifically targeted to pandemic related conditions, and some widely used questionnaires, including an anxiety measure, a depression measure, and a parenting style measure.

### Demographic information and pandemic related questions

This section of the survey collected some basic demographic information, including participants’ gender, whether they were a single child, school, grade, their location, and whether they have participated the online learning. In addition, the survey asked the participants to report whether they have family members or other relatives who were identified as confirmed or suspected COVID-19 cases; whether their parents (or any one of the parents) were quarantined due to the disease; and if any of their members were involved in first-line job responsibilities related to COVID-19.

### Short Egna Minnen Beträffande Uppfostran (S-EMBU)

The Short Egna Minnen Beträffande Uppfostran is a widely used measurement to assess perceived parental rearing style in adolescents [15]. The Chinese version of S-EMBU consists of 23 items and has proven to be a measure with good psychometric properties [8]. The measure has three subscales: Rejection, Emotional Warmth, and Overprotection. For the purposes of this study, we selected a total of 10 items that appear more connected with the pandemic context; each subscale has 3 to 4 items. The items selected included, for example: “I can feel the love from my parents”, “Recently I feel that my parents interfere in everything I do.”, “Recently, my parents often get angry with me for no reasons.” The internal consistency reliability for these three dimensions was moderate, and the total scale of the three dimensions has a Cronbach’s α measurement of 0.813. (Subscale Rejection: Cronbach’s α = 0.780; Subscale Emotional Warmth: Cronbach’s α = 0.770; Subscale Overprotection: Cronbach’s α = 0.562). Calculating the validity of the scale using Confirmatory Factor Analysis (CFA), the Comparative Fit Index (CFI) was 0.962, and the Root Mean Square Error of Approximation (RMSEA) was 0.085, so all the model fit indexes were acceptable.

### Patient Health Questionnaire-9 (PHQ-9)

The Patient Health Questionnaire (PHQ-9 [10];) is a widely used, self-administered instrument intended to assess an individual’s depressive symptoms. The measurement has 9 items, asking individuals to assess their feelings during the past 2 weeks. Sample items included, for example, “Feeling down, depressed, irritable, or hopeless”, and “Trouble concentrating on things like schoolwork, reading, or watching TV”. The scores range from “0” (not at all) to “3” (nearly every day). Total PHQ-9 scores of 5, 10, 15 and 20 represent the cutoffs for mild, moderate, moderately severe, and severe depression, respectively. This means that, in this study, any score higher than 4 was taken as an indication that the individual has depressive symptoms. We therefore used this measure of depression as a dichotomous variable in our study to distinguish individuals with or without depressive symptoms. PHQ-9 has been used with a Chinese population and was found to be a sensitive screening tool with high clinical reliability and validity [26].

### Generalized Anxiety Disorder-7 (GAD-7)

Generalized Anxiety Disorder-7 (GAD-7, [21]) is a well-developed short screening tool designed to assess an individual’s anxiety level for the past 2 weeks. The scale has 7 items, and the response options range from “0” (not at all), to “3” (nearly every day). Sample items included, for example, “Not being able to stop or control worrying”, and “Feeling afraid as if something awful might happen”. Cut points of 5, 10, 15 are interpreted as representing mild, moderate, and severe levels of anxiety. This means that any score higher than 4 indicates the presence of anxiety symptoms. As was the case with the depression measure, we employed a dichotomous variable in this study to distinguish individuals with or without anxiety symptoms. The Chinese version of GAD-7 has been validated with a Chinese population with good reliability and validity [6, 22], and was therefore employed to assess Chinese adolescents’ severity of anxiety.

### Data analysis

An independent sample t-test was used to examine whether there is a clinically significant difference in anxiety, depression, and parental rearing style comparing Wuhan and other urban areas in China (Beijing and Hangzhou, in this case). A Chi-Square test was performed for the categorical variables such as gender, with and without depressive and anxiety symptoms, etc. The significance value was set at *p* < .05 in this study. SPSS Statistics software version 26 was utilized to run this analysis.

The hypothesized moderating and mediating effects were examined by Structural Equation Modeling (SEM) using R version 3.6.1. The goodness of fit was assessed by computing the comparative fit index (CFI), Tucker-Lewis index (TLI), root mean square error of approximation (RMSEA), and standardized root mean residual (SRMR). The acceptable levels of the goodness-of-fit model parameters are CFI > .90, TFI > .90, RMSEA < .05, and SRMR < .05.

## Results

### Location and mental health condition

A Chi-Square test was used for the categorical variables in this study. The descriptive statistics and significance levels were shown in Table 1. The bilateral progressive significance value was set as *p* < .05 in this study. Results showed that there was a statistically significant difference in anxiety symptoms between participants who were from Wuhan compared to other urban areas (χ2_(df = 1)_ = 8.825, *p* = .004), but there was no statistically significant difference in depressive symptoms (χ2_(df = 1)_ = 1.137, *p* = .286). In addition, we did not find significant differences in depression and anxiety symptoms in those who have relatives who participate in epidemic-related work and those whose parents were quarantined due to the disease.

**Table 1:** Descriptive statistics and significance levels of the variables

	Without anxiety symptoms (*N* = 5681) (n%)	With anxiety symptoms (*N* = 2091) (n%)	*P* value
Grade			
Middle School (*n* = 5107)	3936 (69.3%)	1171 (56%)	<.001
High School (*n* = 2665)	1745 (30.7%)	920 (44%)	
Location			
Beijing&Hangzhou (*n* = 4922)	3652 (64.3%)	1270 (60.7%)	0.004
Wuhan (*n* = 2850)	2029 (35.7%)	821 (39.3%)	
Gender			
Male (*n* = 3713)	2823 (49.7%)	890 (42.6%)	<.001
Female (*n* = 4059)	2858 (50.3%)	1201 (57.4%)	
Have Siblings			
No (*n* = 2447)	1716 (30.2%)	731 (35.0%)	<.001
Yes (*n* = 5325)	3965 (69.8%)	1360 (65.0%)	
Relatives COVID work			
No (*n* = 6943)	5079 (89.4%)	1864 (89.1%)	.743
Yes (*n* = 829)	602 (10.6%)	227 (10.9%)	
Relatives Infected			
No (*n* = 7399)	5463 (96.2%)	1936 (92.6%)	<.001
Yes (*n* = 373)	218 (3.8%)	155 (7.4%)	
Parents Quarantined			
No (*n* = 7545)	5527 (97.3%)	2018 (96.5%)	.070
Yes (*n* = 227)	154 (2.7%)	73 (3.5%)	
Study Online			
No (*n* = 1577)	1213 (21.4%)	364 (17.4%)	<.001
Yes (*n* = 6195)	4468 (78.6%)	1727 (82.6%)	
	Without depressive symptoms (*N* = 4438) (n%)	With depressive symptoms (*N* = 3334) (n%)	*P* value
Grade			
Middle School (*n* = 5107)	3178 (71.6%)	1929 (57.9%)	<.001
High School (*n* = 2665)	1260 (28.4%)	1405 (42.1%)	
Location			
Beijing&Hangzhou (*n* = 4922)	2833 (63.8%)	2089 (62.7%)	.286
Wuhan (*n* = 285)	1605 (36.2%)	1245 (37.3%)	
Gender			
Male (*n* = 3713)	2278 (51.3%)	1435 (43%)	<.001
Female (*n* = 4059)	2160 (48.7%)	1899 (57%)	
Have Siblings			
No (*n* = 2447)	1316 (29.7%)	1131 (33.9%)	<.001
Yes (*n* = 5325)	3122 (70.3%)	2203 (66.1%)	
Relatives COVID work			
No (*n* = 6943)	3983 (89.7%)	2960 (88.8%)	.172
Yes (*n* = 829)	455 (10.3%)	374 (11.2%)	
Relatives Infected			
No (*n* = 7399)	4275 (96.3%)	3124 (93.7%)	<.001
Yes (*n* = 373)	163 (3.7%)	210 (6.3%)	
Parents Quarantined			
No (*n* = 7545)	4315 (97.2%)	3230 (96.9%)	.367
Yes (*n* = 227)	123 (2.8%)	104 (3.1%)	
Study Online			
No (*n* = 1577)	979 (22.1%)	598 (17.9%)	<.001
Yes (*n* = 6195)	3459 (77.9%)	2736 (82.1%)	

The cut-off score in this study for with or without anxiety/depressive symptoms is 5

*p* value: Chi-square test for all these categorical variables

### Location and parental rearing styles

When conducting an independent sample t-test of different parental rearing styles and locations (Table 2), we found that all three subscales showed a significant difference between Wuhan and other urban areas in China (Emotional Warmth: t _(df = 7770)_ = 8.254, *p* < .001; Overprotection: t _(df = 7770)_ = − 4.012, *p* < .001; Rejection: t _(df = 7770)_ = − 7.835, *p* < .001). Taking into account the average difference between subscales, the study participants in Wuhan generally consider their parents to be less warm and more overprotective and rejecting.

**Table 2:** Difference of parent rearing style across locations

	Location	n	Average	t	df	Sig.	Average difference	SE difference	Difference 95% confidence interval	
Emotional Warmth				8.254	7770	0	0.401	0.049	0.306	0.496
	Beijing & Hangzhou	4922	9.9	NA	NA	NA	NA	NA	NA	NA
	Wuhan	2850	9.49	NA	NA	NA	NA	NA	NA	NA
Overprotection				−4.012	7770	0	−0.139	0.035	−0.207	−0.071
	Beijing & Hangzhou	4922	3.51	NA	NA	NA	NA	NA	NA	NA
	Wuhan	2850	3.64	NA	NA	NA	NA	NA	NA	NA
Rejection				−7.835	7770	0	−0.367	0.047	−0.459	−0.275
	Beijing & Hangzhou	4922	4.88	NA	NA	NA	NA	NA	NA	NA
	Wuhan	2850	5.25	NA	NA	NA	NA	NA	NA	NA

*NA* Not applicable

### Path analysis results

**Fig. 1:**
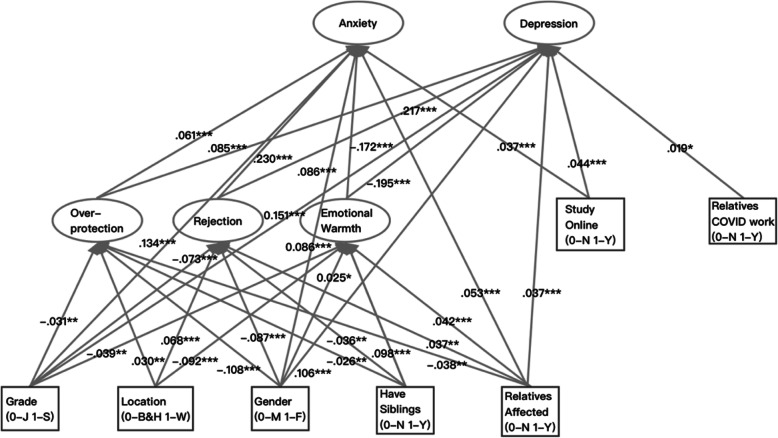
The Path Direction of Variables. Note: The cut-off score in this study for with or without anxiety/depressive symptoms is 5. Std. Est of paths are plotted on the figure. * *p* < .05; ** *p* < .01; *** *p* < .001

The proposed model (Fig. 1) that we tested posits relationships among demographic variables (e.g. grade, location, gender, sibling status), pandemic related information (e.g., relatives who participated in COVID-related work, relatives who were infected), parental rearing styles, and adolescents’ emotional well-being (anxiety and depression). In this model, the comparative fit index (CFI) is 1.000, the Tucker-Lewis index (TLI) is 1.001, the root mean square error of approximation (RMSEA) is 0.000, the standardized root mean residual (SRMR) is 0.003, and the Chi-square is 9.322 (df = 11), resulting in a *p*-value for our model of 0.592. Consequently, in general, the null hypothesis cannot be rejected, as the current model is very close to the real model of our parameters.

However, upon further scrutiny, we found many significant pathways (Table 3). For example, participants’ grade level, gender, a relative being infected, and study online all have direct positive predictive value for depressive and anxiety symptoms, while location and sibling status have indirect predictive value for adolescents’ mental wellbeing. Having relatives who participated in COVID-19 related work was shown to only have positive direct predictive value toward depression, but not anxiety. For three different parental rearing styles, emotional warmth was negatively related to depression and anxiety, but overprotection and rejection had a positive relationship with depression and anxiety. In addition, grade, location, gender, sibling status, and relatives infected in COVID-19 all showed direct predictive value toward the three different parental rearing styles, though they have different directions of relationship. However, among all the model paths that point to anxiety and depression, no matter whether it is a direct or indirect path, the factor of “whether parents are quarantined” in the pandemic related question section is not shown to influence anxiety and depression at a significant level, and it also has no significant relationship with the three subscales of parental rearing styles.

**Table 3:** Total, direct, and indirect relationships between model variables

	With or without depressive symptoms	With or without anxiety symptoms	Emotional Warmth	Overprotection	Rejection
Grade (0-J, 1-S)					
Direct	.151***	.134***	−.039**	−.031**	−.073***
Indirect	−.011*	−.012**	–	–	–
Total	.14***	.122***	–	–	–
Location(0-B&H, 1-W)					
Direct	–	–	−.092***	.03*	.068***
Indirect	.035***	.033***	–	–	–
Total	.035***	.033***	–	–	–
Gender (0-M, 1-F)					
Direct	.106***	.086***	.025*	−.108***	−.087***
Indirect	−.033***	−.031***	–	–	–
Total	.073***	.055***	–	–	–
Have Siblings (0-N, 1-Y)					
Direct	–	–	.098***	−.026*	−.036**
Indirect	−.029***	−.027***	–	–	–
Total	−.029***	−.027***	–	–	–
Relatives COVID Work (0-N, 1-Y)					
Direct	.019*	–	–	–	–
Indirect	–	–	–	–	–
Total	.019*	–	–	–	–
Relatives Infected (0-N, 1-Y)					
Direct	.037***	.053***	−.038**	.042***	.037**
Indirect	.019**	.018**	–	–	–
Total	.056***	.07***	–	–	–
Study Online (0-N, 1-Y)					
Direct	.044***	.037***	NA	NA	NA
Indirect	–	–	NA	NA	NA
Total	.044***	.037***	NA	NA	NA
Emotional Warmth					
Direct	−.195***	−.172***	NA	NA	NA
Indirect	–	–	NA	NA	NA
Total	−.195***	−.172***	NA	NA	NA
Overprotection					
Direct	.085***	.061***	NA	NA	NA
Indirect	–	–	NA	NA	NA
Total	.085***	.061***	NA	NA	NA
Rejection					
Direct	.217***	.23***	NA	NA	NA
Indirect	–	–	NA	NA	NA
Total	.217***	.23***	NA	NA	NA

*NA* Not applicable

Std.Est of paths were represented. * *p* < .05; ** *p* < .01; *** *p* < .001, insignificant paths were represented as ‘-’

## Discussion

This study echoed some previous studies’ results but also discovered some novel ones that are closely connected with COVID-19. To answer the research questions, we will first focus on whether location make a difference. Then, we will discuss other predictive factors presented in this research.

### Does location matter?

Wuhan is a city that attracted the attention of the Chinese people and the rest of the world during COVID-19. As of April 24, 2020, statistics have shown that there were 3869 deaths, over 50,333 COVID-19 diagnoses, and a mortality rate of 7.69% in Wuhan, which is much higher than the rest of China (mortality rate of 0.8%) [19]. A previous study found that adolescents’ depressive symptoms in Wuhan were significantly higher than in other cities in Hubei Province, which indicated that the severity of infection is likely impacting the depressive symptoms [27]. However, our study found that there is no statistically significant difference in adolescents’ depression in Wuhan, but there is a statistically significant difference in anxiety, compared to other urban areas in China. This means that Wuhan’s adolescents appeared to be more anxious than those in other urban areas, but their depression level seems to be similar when compared regionally. This result is supported by Jiao and his research team [9]; they found that that even though the rate of negative emotions was likely to be higher in children living in highly epidemic areas, the difference between areas did not reach a significant level. However, the more in-depth analysis in our study indicated that, even though there is no significant direct effect and difference, by investigating the influence of the parental rearing style, regional factors have certain indirect effects on adolescents’ depression and anxiety.

In our study, we found that Wuhan adolescents’ parents were less warm and supportive, more protective, and showed more emotional rejection toward their children compared to other urban areas. This might be related to parents’ higher distress during the COVID-19 period in Wuhan. A previous study found that parenting stress mediates between a child’s temperament and parenting style [14]; another study that investigated children with developmental disabilities found that, for parents who are under higher stress, it is very difficult for them to implement authoritative parenting approaches, an approach that combines warmth, sensitivity, and limits setting [25]. Therefore, it is understandable that during times of stress and uncertainty, parenting styles may change accordingly. In the context of COVID-19, parents are faced with greater financial pressure, which might lead to bad temper and an overreliance on less effective parenting approaches. As a result, the relationship between parents and children may be strained by the outbreak. Correspondingly, the psychological problems of parents during the epidemic might also lead to the decline of parents’ emotional warmth and support to their children and a stronger emotional response to children’s misbehavior, which will eventually lead to the escalation of conflicts between children and their parents [17], and which would subsequently have a negative impact on adolescents’ mental health. These parental rearing factors, therefore, indirectly and positively impact adolescents’ depression and anxiety symptoms compared to other urban areas. This finding echoes previous findings [23, 30], in which the warmth and support that parents showed to their children were found to usually protect their children from feeling depressed or anxious; but for parents who were harsh and relied excessively on punishment with their children, the anxiety and depression on the latter would increase correspondingly.

### Other important risk factors for adolescents’ mental health condition during the COVID-19

Results from this study also showed some risk factors. First of all, higher grades (high school compared to middle school in this case), and female gender, are two risk factors for adolescents. This finding is supported by the research of Zhou and his team [32], in which these two factors were also found to be risk factors for depressive and anxiety symptoms during the COVID-19 pandemic. In addition, there were similar findings in several previous studies. For instance, Branje, Hale III, Frijns and Meeus [1] stated that older adolescents reported more depressive symptoms; moreover, their study connected adolescents’ depressive symptoms with their relationship with parents and found that depressive adolescents appear to have lower quality relationship with their parents. It might also be the case that the isolation brought on by the pandemic has a differential effect on older children because their independence is more compromised (i.e., younger kids are developmentally more naturally dependent upon their parents).

Another interesting risk factor is the only child status. Even though a previous study indicated that, under the one-child policy, children with siblings in China have higher anxiety and depression than those who are only children [28], during the COVID-19 period, it appears that having siblings became a protective factor, possibly due to their reduced sense of isolation. Our research showed that adolescents who are the only child at home are more likely to perceive their parents as overprotective toward them during the pandemic. This therefore relates to more depressive and anxious symptoms. This is understandable due to the challenges that COVID-19 brought to the family system. To name a few of the challenges, parents’ relationships to their children were impacted in this time period, and adolescents were isolated from their peers and other supportive adults. Thus, preservation of the sibling relationship appears to have become a protective factor for adolescents’ mental health [17], where adolescents can feel less lonely and they can be more supportive of each other.

Finally, a family member’s compromised health condition is a risk factor that should not be ignored. There is convincing evidence that adversity in family social context negatively influences children’s adjustment [20]. Our study supported this finding and provided evidence that, when there is a family member who was infected with COVID-19, it not surprisingly positively predicted adolescents’ anxiety or depressive symptoms. Even though the effect size is small, the impact on adolescents’ mental health condition should not be ignored. Similar results were found by Cao and his team [2]; when they examined a group of college students, they discovered that, for those students who had relatives infected with COVID-19, there was an increase in their anxiety levels.

### Limitations and implications

This study also has some limitations that are important to note. First, we chose Beijing and Hangzhou as two main eastern cities to represent other urban areas in China, not including rural areas. This might have created a biased sample that cannot adequately represent the entire Chinese adolescent population. Second, for the purpose of shortening the survey questionnaire, we selected 10 items from the S-EMBU. Although we have examined the construct validity of this shortened version, it is less rigorous compared to using the full scale and collecting information about mother and father’s parenting style separately. Third, we are uncertain if the parenting style difference between Wuhan and other urban areas (Beijing and Hangzhou in this case) is related to the severity of this pandemic. Therefore, we recommend conducting a study after COVID-19 to examine the parenting style differences among different regions in China. In addition, we found that studying online only has a direct effect on adolescents’ mental health condition, but not an indirect effect through parenting on the outcomes. This result might be related to the lack of information collected regarding students’ online study conditions. We did not differentiate between whether students were taking online courses due to home quarantine policy implemented during COVID-19, or because they were having online class as a part of their regular routine and they happen to continue this form of study during the pandemic. Also, when we collected data in late February and early March, online learning had just started and not all students were involved in this type of learning, therefore, its impact on parenting style may have not emerged yet. Thus, we recommend that researchers further investigate online study and its impact on parents’ rearing style and adolescents’ mental health, since online learning might become an increasingly important way of receiving knowledge in the future. Lastly, how adolescents perceive their parents’ rearing style might be related to adolescents’ own level of stress, which was not measured in this study. Researchers can investigate this variable in the future and examine whether adolescents’ stress predicts their perception of their parents’ behavior.

Based on the results of this study, there are several implications for mental health professionals, parents, and teachers. First, parents, especially for parents in the Wuhan area, it is important to be aware of their parental rearing style and how children perceive their style of parenting. When facing certain stressors, parents might change their reaction toward their children; when children perceive their parents to be more harsh, overprotective, or show more emotional rejection toward them, their mental health condition might be compromised. This means that parents should explore ways to take care of themselves emotionally, find more coping resources during this challenging time period, and try to show more emotional warmth towards their children. Second, clinicians can provide intervention for parents and clarify whether the change of parenting style can change adolescents’ anxiety and depression level. Furthermore, stakeholders should pay particular attention to adolescents who are female, in higher grades, who are the only child in the family, and whose family members got infected during the pandemic. These factors are all risk factors that were found in this study, and might put these adolescents at higher risk than others.

## Conclusions

Adolescents comprise a population that might be exposed to some level of risk during COVID-19. Our study, based upon a fairly large sample size, uncovered a new finding that was not found in previous studies. We found that Wuhan adolescents’ anxiety symptoms were significantly higher than in other urban areas, but not their depressive symptoms, which partially speaks to Chinese adolescents’ resilience in the face of this overwhelming public health event. Nevertheless, Wuhan adolescents’ parents might be under higher stress than other urban areas, and that, in turn, would have a negative effect on the outcome of some adolescents’ emotional state. Therefore, we should not only pay attention to adolescents’ emotional well-being and the risk factors, but also take their parents’ mental health condition into consideration and provide intervention from a systemic level.

## Data Availability

The datasets used and/or analyzed during the current study are available from the corresponding author upon request.
